# Supplementary material for: Genetic drift from the out-of-Africa bottleneck leads to biased estimation of genetic architecture and selection
Source: Eur J Hum Genet. 2021 Apr 13;29(10):1549–56. doi: 10.1038/s41431-021-00873-2 (PMC8484570; doi:10.1038/s41431-021-00873-2)

Supplementary Information for: Genetic drift from the out-of-Africa bottleneck leads to biased estimation of genetic architecture and selection

Bilal Ashraf[1,2] and Daniel John Lawson[1,3]*

[1]: Department of Statistical Sciences, School of Mathematics, University of Bristol, Fry Building, BS8 1UG, UK

[2]: Department of Anthropology, Durham Research Methods Centre, Dawson Building, University of Durham, DH13LE, UK

[3]: Integrative Epidemiology Unit, Population Health Sciences, University of Bristol, Oakfield House, Bristol, BS8 2BN, UK

* Corresponding author: dan.lawson@bristol.ac.uk

Supplementary Figure 1

Estimates of genetic variance with different thresholds for all population groups at S = -1, as reported in Figure 2 in the form of Heritability.

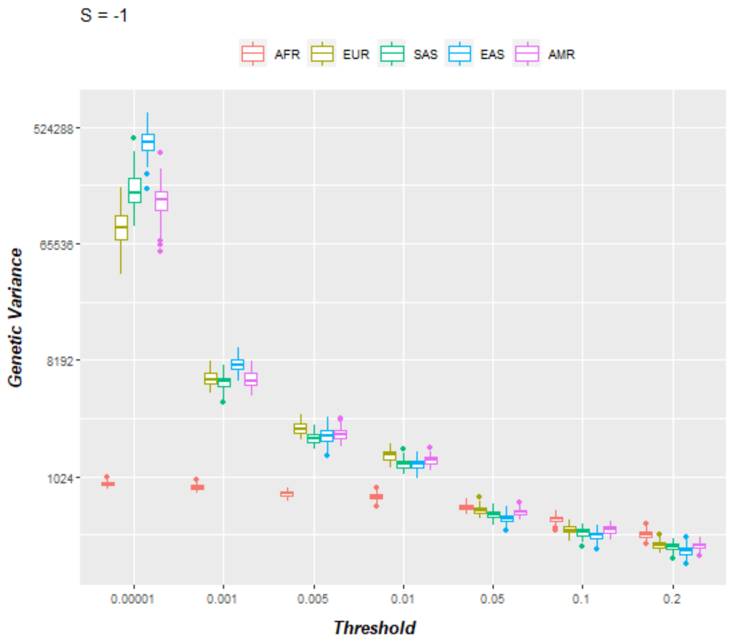

Supplementary Figure 2

Estimates of genetic variance with different thresholds for all population groups at S = -0.5, as reported in Figure 2 in the form of Heritability.

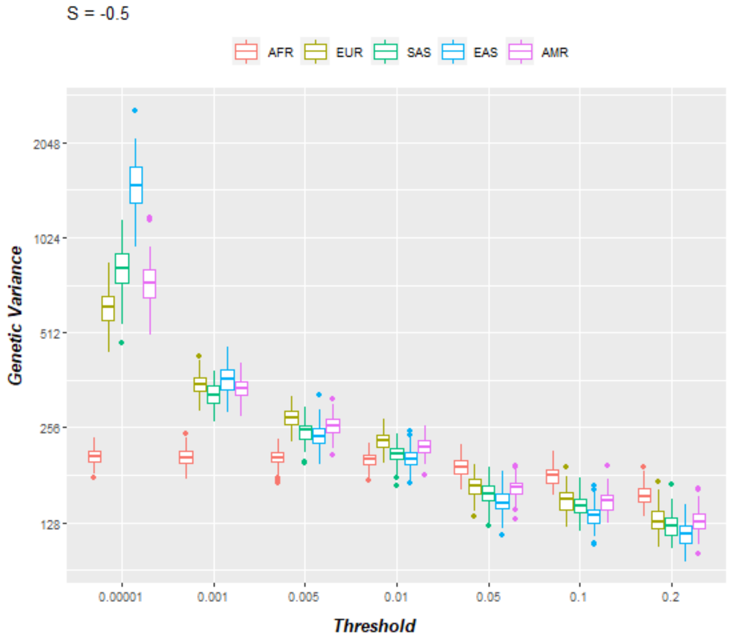

Supplementary Figure 3

Simulation of Genetic architecture with Genetic Drift at varied values of S (Assuming $F_{st} = 0.2$). The model and presentation follows Figure 1, and varies only by a) showing a larger range of quantiles, and b) shows S=-1,-0.5,-0.25,0. The number of SNPs required for drift to result in a large effect SNP that dramatically changes model inference is of the order 1/(1-q) where q is the first quantile for which the drifted model has significant mass above the corresponding undrafted curve.

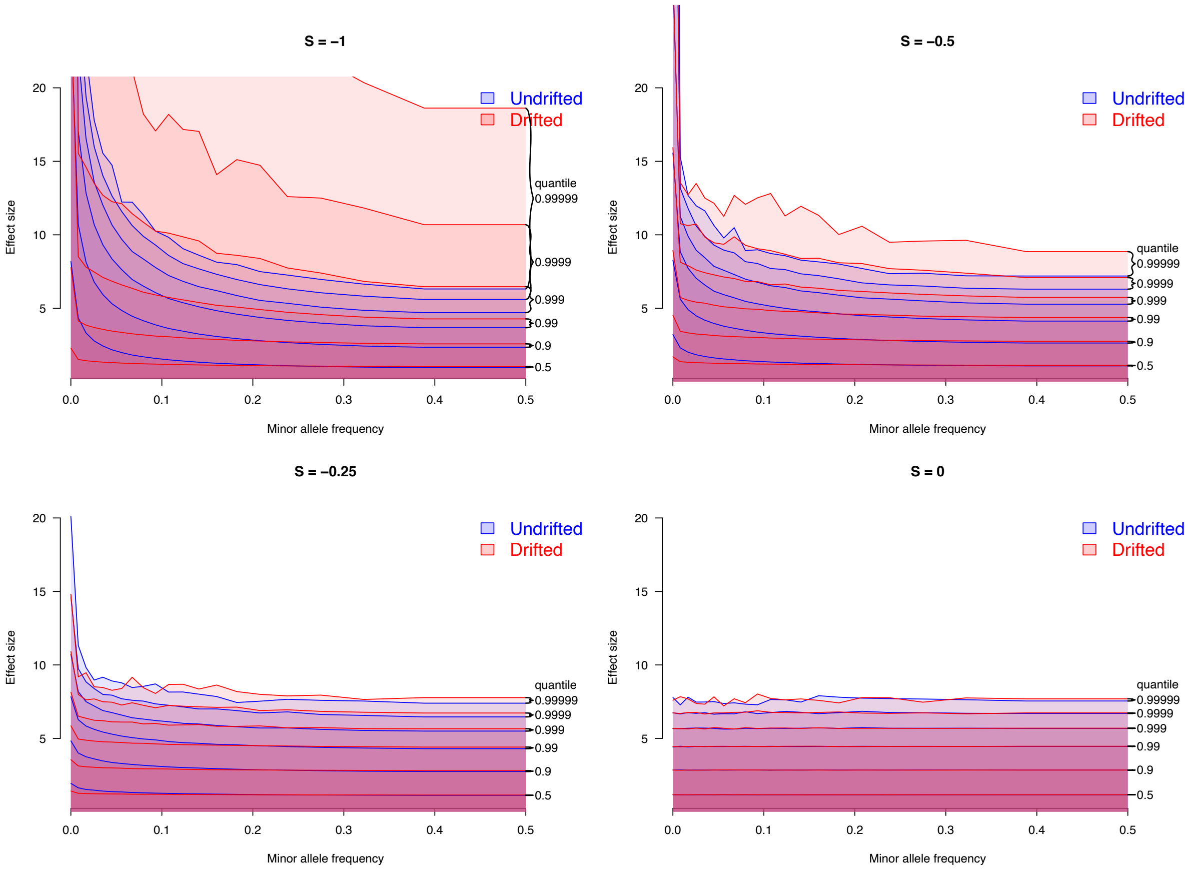

Supplementary Figure 4

Impact of archaic admixture on Genetic architecture, assuming $F_{st} = 0.1$, comparing (left) Genetic drift from the Out-of-Africa event only, to (right) additionally adding 5% of the genome having $F_{st} = 0.5$.

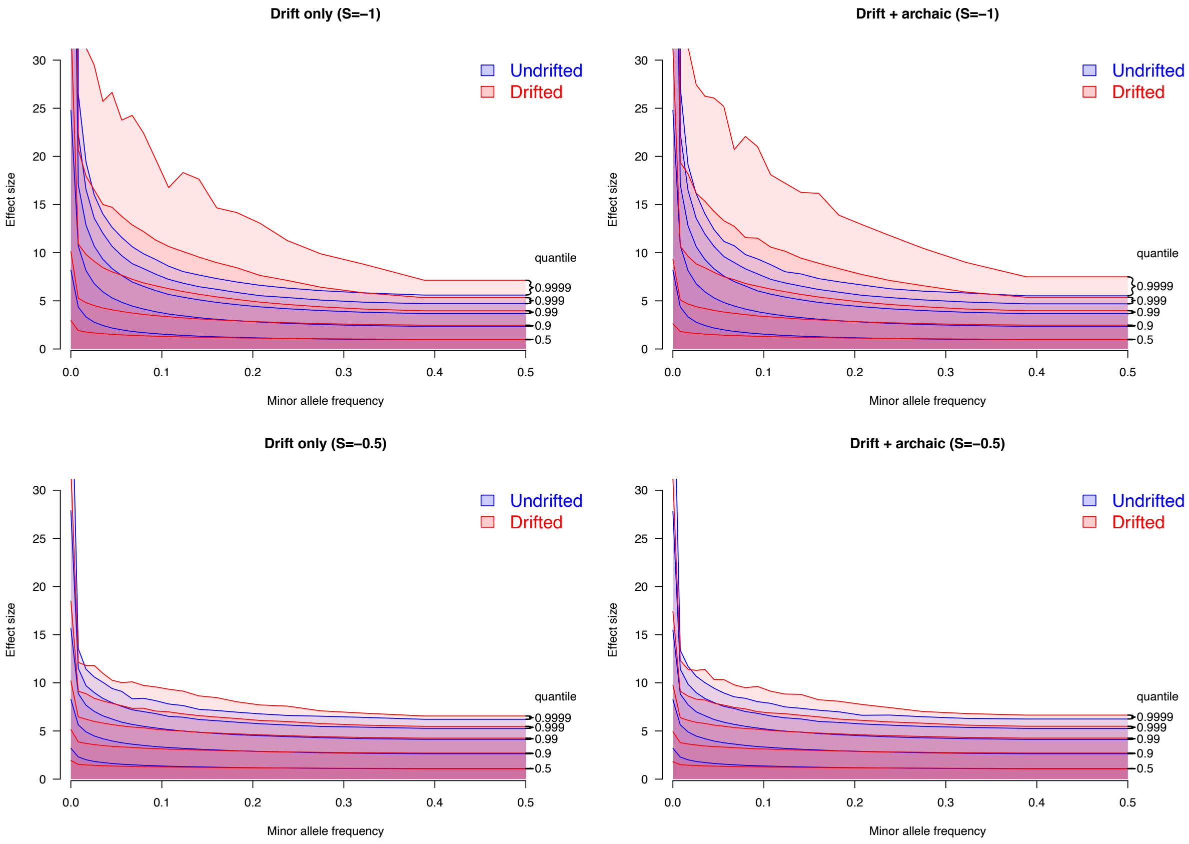

Supplement: Supplementary file 1 — Supplementary Material [file 41431_2021_873_MOESM1_ESM.pdf]
